# *O*-Desmethyltramadol Enhanced Anti-Cancer Efficacy over Tramadol Through Non-μ-Opioid Receptor and Differential Cellular Contexts of Human Breast Cancer Cells

**DOI:** 10.3390/ijms26094139

**Published:** 2025-04-27

**Authors:** Zih-Syuan Wu, Yi-Hsuan Huang, Shih-Ming Huang

**Affiliations:** 1 Graduate Institute of Life Sciences, National Defense Medical Center, Taipei City 114, Taiwan; g8401011@gmail.com; 2 Department of Anesthesiology, Tri-Service General Hospital, National Defense Medical Center, Taipei City 114, Taiwan; 3 Department of Biochemistry, National Defense Medical Center, Taipei City 114, Taiwan

**Keywords:** Tramadol, *O*-desmethyltramadol, breast cancer, cytotoxicity, μ-opioid receptor, ER stress, tumor microenvironment

## Abstract

Tramadol, a widely used analgesic, has recently been explored for its potential anti-cancer effects. However, the antitumor dosage of tramadol is over its current clinical application. Its primary metabolite, *O*-desmethyltramadol, has greater μ-opioid receptor affinity and stronger pharmacological activity. Hence, we sought to examine whether the cytotoxic effect of *O*-desmethyltramadol was better than tramadol on breast cancer cells. Our results showed that *O*-desmethyltramadol significantly reduced cell viability in breast cancer cells, with IC50 values of 64.2 μg/mL (MDA-MB-231) and 96.7 μg/mL (MCF-7), demonstrating over ten-fold greater potency than tramadol. The presence of a μ-opioid receptor antagonist Alvimopan did not alter the cytotoxic effects of tramadol and *O*-desmethyltramadol, indicating a non-opioid receptor-mediated mechanism. Compared with antitumor activity of tramadol mediated through ER stress, we confirmed that *O*-desmethyltramadol induced ER stress proteins, including the p-eIF2α/eIF2α ratio, ATF4, and CHOP. In MDA-MB-231 cells, *O*-desmethyltramadol treatment elevated mRNA expression levels of *ATF4*, *CHAC1*, and *DDIT3* by approximately 2-fold. In MCF-7 cells, the induction was even more pronounced, with *ATF4* increased 1.7-fold, *CHAC1* 12-fold, and *DDIT3* 9-fold. Beyond the opioid receptor-mediated pathway, we further analyzed the differential functions of *O*-desmethyltramadol than tramadol using the RNA-seq analysis. The pathway enrichment analyses revealed that *O*-desmethyltramadol influenced immune and inflammatory pathways, such as TNF and IL-6/JAK/STAT3 signaling in MDA-MB-231 cells, while in MCF-7 cells, it affected metabolic and transcriptional pathways, including mTOR and MAPK signaling. Gene Set Enrichment Analysis further highlighted *O*-desmethyltramadol’s role in interferon response and tumor microenvironment modulation. Four upregulated genes and five downregulated genes were modulated by *O*-desmethyltramadol in MDA-MB-231 and MCF-7 cells. Overall, our findings indicated that *O*-desmethyltramadol exerted potent anti-cancer effects through multiple non-opioid mechanisms, with distinct response from tramadol depending on breast cancer subtype. These findings not only highlight the therapeutic potential of *O*-desmethyltramadol as a novel adjunct in breast cancer treatment, but also emphasize the need for further investigation into its safety and clinical applicability in oncology.

## 1. Introduction

Tramadol is a widely utilized analgesic in clinical practice, commonly prescribed for the management of moderate cancer-related and postoperative pain. Its molecular structure contains two chiral carbon atoms, and the commercially available formulation is a racemic mixture consisting of equal proportions of the (+)-1R,2R and (−)-1S,2S enantiomers [1]. In humans, tramadol undergoes extensive hepatic metabolism. The primary metabolic pathways—*O*-desmethylation and *N*-desmethylation—are mediated by cytochrome P450 isoenzymes, including cytochrome P450 2D6 (CYP2D6), cytochrome P450 2B6 (CYP2B6), and cytochrome P450 3A4 (CYP3A4) [2]. This metabolic process produces at least 23 metabolites, among which *O*-desmethyltramadol stands out as the pharmacologically active metabolite. *O*-desmethyltramadol exhibits a significantly higher affinity for the μ-opioid receptor compared to the parent compound, contributing to its enhanced analgesic potency [3]. The *O*-desmethyltramadol metabolite is approximately six times more potent than tramadol itself in providing analgesia and is estimated to be 300 times more effective at binding to the μ-opioid receptor than the parent drug [4]. Tramadol achieves its analgesic effects through two complementary and synergistic mechanisms: activation of the μ-opioid receptor and inhibition of neurotransmitter reuptake [5]. The opioid receptor-mediated analgesia primarily stems from the active metabolite, *O*-desmethyltramadol, while the parent drug is responsible for inhibiting the reuptake of neurotransmitters such as serotonin and norepinephrine. This dual action enhances the suppression of pain transmission in the spinal cord, providing a robust analgesic effect.

Ensuring the safe and effective use of opioids in oncology is essential for providing adequate pain management to cancer patients. Opioids mediate their analgesic effects by binding to specific receptors, including μ, κ, δ, and opioid-like receptors. Among these, the μ-opioid receptor is the most studied in the context of cancer, being expressed both pre- and post-synaptically and playing a pivotal role in modulating nociceptive signaling [6]. Upon receptor activation, opioids reduce the depolarization of afferent nociceptive neurons, thereby inhibiting pain transmission [7]. Interestingly, μ-opioid receptor expression has been identified in several tumor types, and elevated μ-opioid receptor levels have been associated with an increased risk of lymph node metastasis [8]. These findings have prompted investigations into the potential role of opioids not only as analgesics but also as modulators of tumor biology. A growing body of preclinical and clinical evidence has examined the relationship between opioid use and cancer progression. For example, chronic morphine treatment has been shown to attenuate cell growth in BT474 human breast cancer cells by altering the ErbB signaling network [9]. Moreover, opioid ligands targeting various receptor subtypes have demonstrated dose-dependent growth inhibition in MCF-7 breast cancer cells, an effect that was reversed by co-treatment with the opioid receptor antagonist naloxone [10]. Clinically, studies have also suggested a potential protective role of intraoperative opioid use in improving recurrence-free survival in triple-negative breast cancer (TNBC) patients. This effect may be partly explained by the expression patterns of opioid receptors, where protumorigenic receptors are either downregulated or absent, while antitumorigenic receptors are upregulated [11].

Recent studies have highlighted that tramadol not only serves as an effective analgesic but also demonstrates significant potential as an anti-cancer agent, particularly in combating breast cancer. Evidence from both in vitro [12] and in vivo [13] research has revealed that tramadol exerts anticancer effects on breast cancer cells. Notably, clinical reports suggest that the postoperative use of tramadol is associated with a reduced risk of breast cancer recurrence, further emphasizing its potential therapeutic value [14]. In our previous research, we demonstrated that tramadol inhibited cell migration, colony formation, and invasion in a dose-dependent manner across two breast cancer cell lines [15]. Additionally, when used in combination with doxorubicin, a widely used chemotherapeutic agent, tramadol exhibited synergistic anticancer effects. These synergistic interactions were observed in MDA-MB-231 cells, a model for TNBC, and in MCF-7 cells, which represent estrogen receptor-positive breast cancer. This combination not only enhanced anticancer efficacy but also suggested that tramadol may serve as sensitizer against breast cancer to reduce the side effects and recurrent rates of current therapeutics. Nonetheless, these anti-cancer effects were observed only at concentrations exceeding those used in clinical analgesia, thereby limiting the feasibility of tramadol as a therapeutic agent in oncology.

Given that *O*-desmethyltramadol is the primary active metabolite of tramadol, with significantly higher μ-opioid receptor affinity and enhanced pharmacological potency, we hypothesized that it may exhibit greater cytotoxic activity than tramadol in breast cancer cells. Importantly, we aimed to investigate whether these effects were dependent on μ-opioid receptor signaling or involved alternative pathways. By comparing the cytotoxic and mechanistic effects of tramadol and *O*-desmethyltramadol, including transcriptomic analysis through RNA-seq, we sought to uncover whether *O*-desmethyltramadol could serve as a more potent and mechanistically distinct anti-cancer agent. This comprehensive analysis will provide valuable insights into the potential anti-cancer mechanisms of *O*-desmethyltramadol and tramadol, paving the way for their potential application in oncology.

## 2. Results

### 2.1. O-Desmethyltramadol Had a Better Effect than Tramadol in Inhibiting the Cell Viability of Breast Cancer Cells

**Figure 1:**
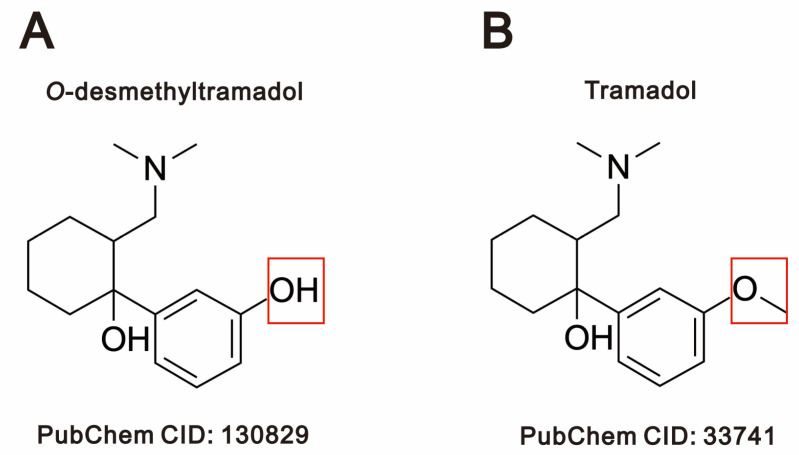
A diagram of (**A**) *O*-desmethyltramadol and (**B**) tramadol. Compound identification (CID) is the identifier from a database of chemical molecules and their activities of biological assays in PubChem.

*O*-desmethyltramadol is the main active metabolite of tramadol, formed through *O*-desmethylation by the CYP2D6 [2]. The key distinction between *O*-desmethyltramadol (Figure 1A) and tramadol (Figure 1B) is illustrated.

**Figure 2:**
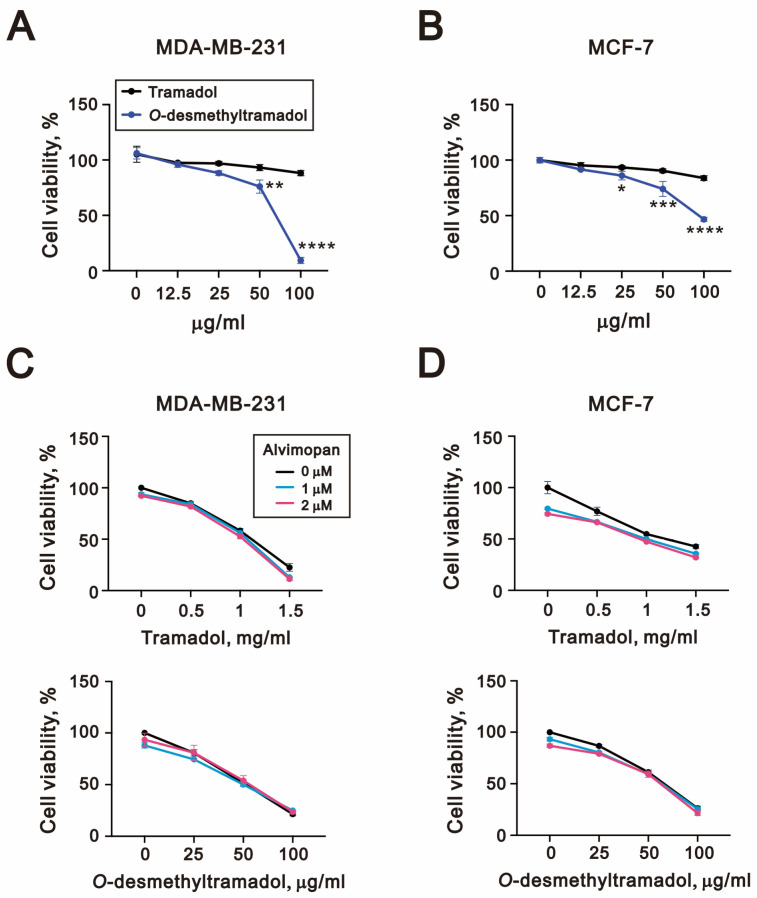
*O*-desmethyltramadol was more effective than tramadol in suppressing breast cancer cell viability, independent of opioid receptor activation. (**A**) MDA-MB-231 and (**B**) MCF-7 cells were treated with 0, 12.5, 25, 50, and 100 μg/mL tramadol or *O*-desmethyltramadol for 48 h. Cell viability was determined by CellTiter-Glo assay. Two-way ANOVA with Dunnett’s multiple comparisons was performed, and the results were compared with the vehicle group. * *p* < 0.05, ** *p* < 0.01, *** *p* < 0.001, and **** *p* < 0.0001. (**C**) MDA-MB-231 and (**D**) MCF-7 cells were pretreated with 0, 1, or 2 μM alvimopan for 2 h, followed by treatment with tramadol (0, 0.5, 1, or 1.5 mg/mL) or *O*-desmethyltramadol (0, 25, 50, or 100 μg/mL) for 48 h. Cell viability was determined by CellTiter-Glo assay.

First, we compared whether tramadol and its metabolite, *O*-desmethyltramadol, affected the cell viability of breast cancer cells. The results demonstrated that *O*-desmethyltramadol markedly reduced cell viability in a dose-dependent manner in MDA-MB-231 cells, with its effects becoming especially prominent at lower concentrations (50 µg/mL and 100 µg/mL), yielding a half maximal inhibitory concentration (IC50) value of 64.2 µg/mL (Figure 2A). Similarly, in MCF-7 cells, *O*-desmethyltramadol exhibited a significantly stronger cytotoxic effect compared to tramadol, with noticeable reductions in cell viability occurring at concentrations as low as 25 µg/mL and an IC50 of 96.7 μg/mL (MCF-7) (Figure 2B). Based on the data presented in Figure 2C,D, the calculated IC50 values of tramadol were approximately 1083 µg/mL for MDA-MB-231 cells and 1184 µg/mL for MCF-7 cells.

Next, we investigated whether the μ-opioid receptor antagonist alvimopan influenced the ability of tramadol and *O*-desmethyltramadol to reduce cell viability in MDA-MB-231 and MCF-7 cells. However, the addition of alvimopan did not significantly alter the reduction in cell viability caused by tramadol or *O*-desmethyltramadol in either cell line, suggesting that the observed effects are mediated through a μ-opioid receptor-independent mechanism (Figure 2C,D).

### 2.2. O-Desmethyltramadol Triggered ER Stress in Breast Cancer Cells

**Figure 3:**
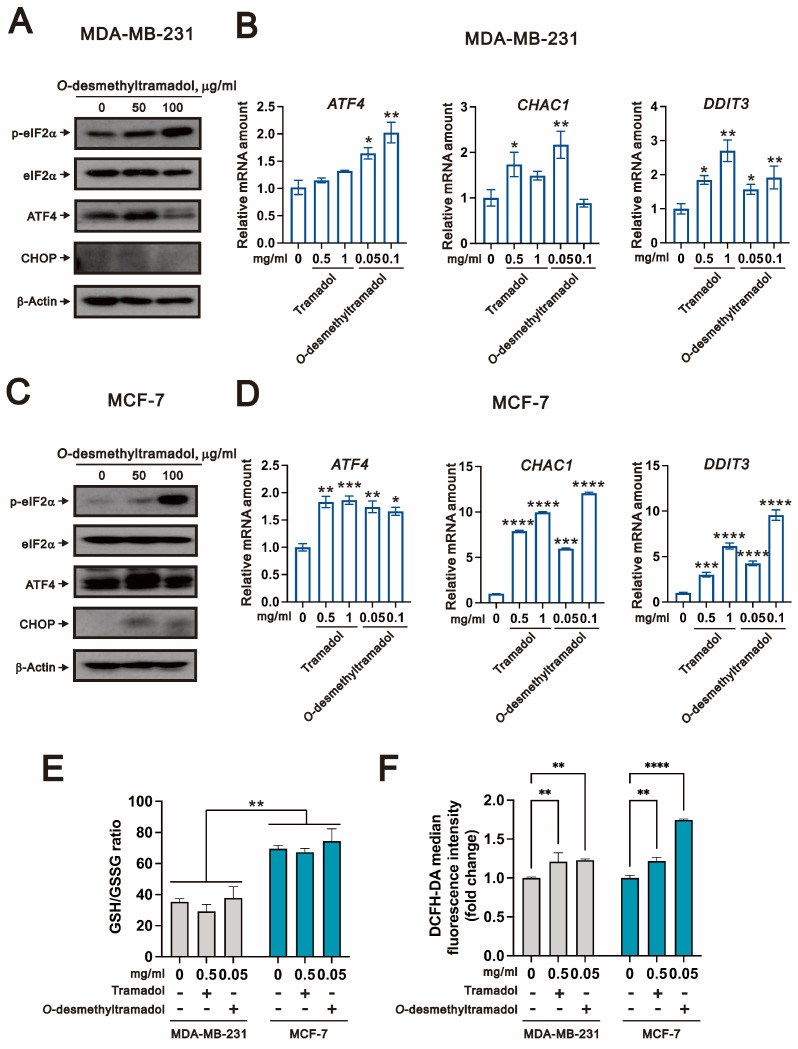
*O*-desmethyltramadol-induced ER stress in breast cancer cells. (**A**,**C**) MDA-MB-231 and MCF-7 cells were treated with 0, 50, and 100 μg/mL of *O*-desmethyltramadol for 4 h. After treatment, the cells were collected and analyzed by western blotting, with β-actin used as an internal control. (**B**,**D**) MDA-MB-231 and MCF-7 cells were treated with 0, 0.5, and 1 mg/mL tramadol or 0.05 and 0.1 mg/mL *O*-desmethyltramadol for 4 h, and the cells were then collected for qPCR analysis, with β-actin used as an internal control. Bars represent the mean ± SEM of three independent experiments. One-way ANOVA followed by Tukey’s multiple comparisons test was performed, and the results were compared with the vehicle group. * *p* < 0.05, ** *p* < 0.01, *** *p* < 0.001, **** *p* < 0.0001. (**E**) MDA-MB-231 and MCF-7 cells were treated with 0 or 0.5 mg/mL tramadol, or 0.05 mg/mL *O*-desmethyltramadol for 4 h. Following treatment, cells were harvested, and intracellular GSH/GSSG levels were measured using the GSH/GSSG-Glo™ Assay. Bars represent the mean ± SEM of three independent experiments. Two-way ANOVA with Dunnett’s multiple comparisons was performed, and the results were compared with the vehicle group. ** *p* < 0.01. (**F**) MDA-MB-231 and MCF-7 cells were treated with 0 or 0.5 mg/mL tramadol, or 0.05 mg/mL *O*-desmethyltramadol for 4 h. Following treatment, cells were harvested, and intracellular ROS levels were quantified using DCFH-DA staining. Bars represent the mean ± SEM of three independent experiments. Two-way ANOVA with Dunnett’s multiple comparisons was performed, and the results were compared with the vehicle group. ** *p* < 0.01, **** *p* < 0.0001.

Since our previous research established that tramadol induces endoplasmic reticulum stress (ER stress) through the p-eIF2α (phosphorylation of the eukaryotic initiation factor 2)/ATF4 (activating transcription factor 4)/CHOP (C/EBP homologous protein) signaling axis in breast cancer cells, we aimed to investigate whether *O*-desmethyltramadol, the active metabolite of tramadol, also triggers ER stress in these cells. To assess this, we examined the expression levels of key ER stress markers using western blot analysis. Our results demonstrated that treatment with 50 μg/mL of *O*-desmethyltramadol led to an increase in the protein expression of both p-eIF2α/eIF2α ratio and ATF4 in MDA-MB-231 cells, indicating activation of the ER stress pathway (Figure 3A). At a higher concentration of 100 μg/mL, p-eIF2α/eIF2α ratio expression continued to increase, whereas ATF4 expression decreased, suggesting a concentration-dependent regulatory effect.

To further confirm these observations at the transcriptional level, we conducted a quantitative polymerase chain reaction (qPCR) to assess the expression of ATF4 and its well-established downstream target genes involved in cellular stress responses, including *glutathione-specific gamma-glutamylcyclotransferase 1* (*CHAC1*) and *DNA damage-inducible transcript 3* (*DDIT3*). The results demonstrated that only *O*-desmethyltramadol significantly induced *ATF4* mRNA expression in a dose-dependent manner, while tramadol did not show this effect in MDA-MB-231 cells (Figure 3B). For downstream targets, *CHAC1* was significantly upregulated at 0.5 mg/mL of tramadol and 0.05 mg/mL of *O*-desmethyltramadol, while *DDIT3* displayed a dose-dependent and significant increase in expression following treatment with either tramadol or *O*-desmethyltramadol in MDA-MB-231 cells (Figure 3B). In MCF-7 cells, treatment with 50 μg/mL of *O*-desmethyltramadol significantly induced the protein expression of ATF4 and CHOP, while 0.1 mg/mL of *O*-desmethyltramadol further upregulated the p-eIF2α/eIF2α ratio (Figure 3C). At the transcriptional level, both tramadol and *O*-desmethyltramadol significantly induced *ATF4* mRNA expression in MCF-7 cells. For downstream genes, both *CHAC1* and *DDIT3* were significantly upregulated in a dose-dependent manner by Tramadol and *O*-desmethyltramadol (Figure 3D).

Here, tramadol and *O*-desmethyltramadol elevated the expression of *CHAC1* mRNA in MDA-MB-231 and MCF-7 cells. A recent study showed that CHAC1 degradation of GSH (glutathione) enhances cystine-starvation-induced necroptosis and ferroptosis through the activated GCN2 (general control nonrepressed 2)-eIF2α-ATF4 pathway in TNBC cells [16]. To further investigate, we analyzed the GSH/GSSG (oxidized glutathione) ratio in MDA-MB-231 and MCF-7 cells following treatment with tramadol and *O*-desmethyltramadol. Our data showed that MCF-7 cells exhibited higher GSH/GSSG ratio than MDA-MB-231 cells (Figure 3E), but no significant differences in the GSH/GSSG ratio were observed after treatment with either tramadol or *O*-desmethyltramadol in MDA-MB-231 and MCF-7 cells. In our previous work, tramadol was showed to elevated cytosolic reactive oxygen species (ROS) levels in human endometrial cancer cells [17]. Therefore, we next assessed the impact of tramadol and *O*-desmethyltramadol on ROS levels in breast cancer cells using 2′,7′-dichlorodihydrofluorescein diacetate (DCFH-DA) staining. Our results confirmed that both tramadol and *O*-desmethyltramadol significantly increased intracellular ROS levels in MDA-MB-231 and MCF-7 cells (Figure 3F). Collectively, these findings suggest that the induction of *CHAC1* expression by tramadol and *O*-desmethyltramadol may be mediated through elevated ROS levels rather than changes in glutathione redox balance, highlighting a potential oxidative stress-related mechanism underlying their effects in breast cancer cells.

### 2.3. Compared to Tramadol, O-Desmethyltramadol Exerts Distinct Effects on Different Types of Breast Cancer Cells by Modulating Various Functional Pathways

**Figure 4:**
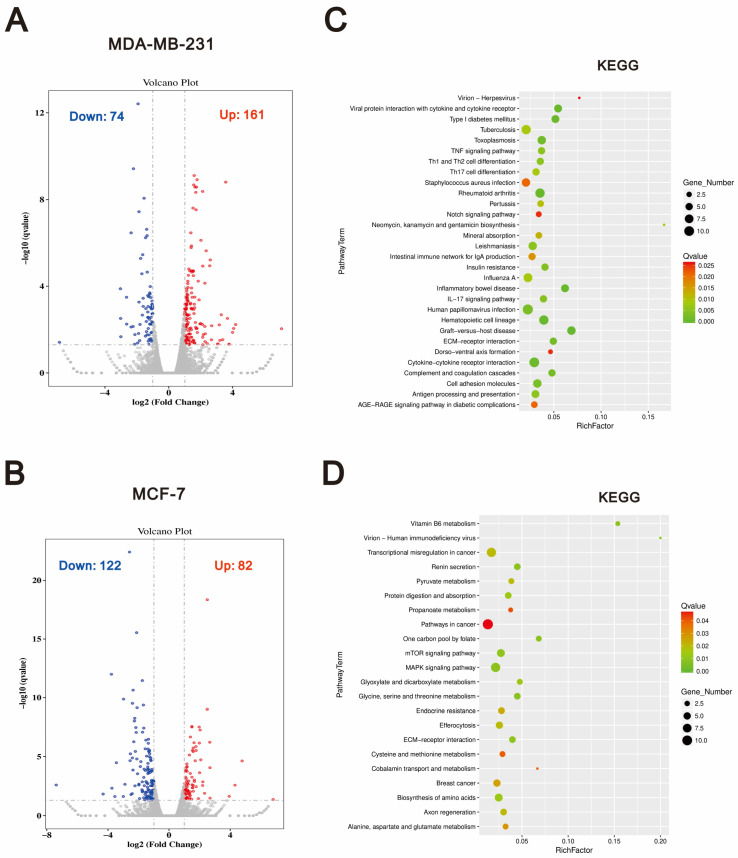
*O*-desmethyltramadol showed different effects on different breast cancer cell types compared to tramadol, modulating several signaling pathways. (**A**,**B**) A volcano plot illustrating the differentially expressed genes in MDA-MB-231 and MCF-7 cells following treatment with 0.05 mg/mL *O*-desmethyltramadol compared to 0.5 mg/mL tramadol. Genes with higher expression in *O*-desmethyltramadol-treated cells are represented by red dots, while blue dots denote genes with reduced expression. The *Y*-axis represents −log_10_ *p* values, and the *X*-axis displays log_2_ fold change values. (**C**,**D**) A scatter plot showing KEGG pathway enrichment statistics in MDA-MB-231 and MCF-7 cells following treatment with 0.05 mg/mL *O*-desmethyltramadol compared to 0.5 mg/mL tramadol. The rich factor represents the ratio of differentially expressed genes annotated to a specific pathway term to the total number of genes annotated to that pathway. A higher rich factor indicates greater enrichment. The Q-value is the adjusted *p*-value, ranging from 0 to 1, with lower values indicating higher significance.

To gain a more comprehensive understanding of the different mechanisms by which tramadol and *O*-desmethyltramadol induce cell death in breast cancer cells, we conducted RNA sequencing (RNA-seq) analysis. This approach allowed us to investigate global gene expression changes and identify key signaling pathways that may contribute to the distinct effects of *O*-desmethyltramadol and tramadol on breast cancer cell viability. To visualize the transcriptional differences between *O*-desmethyltramadol and tramadol treatment, RNA-seq results were presented as a volcano plot. In this plot, significantly upregulated genes are highlighted in red, while downregulated genes are shown in blue, illustrating the distinct gene expression profiles induced by the two compounds (Figure 4A,B). To further investigate the biological significance of these gene expression changes, we performed KEGG (Kyoto Encyclopedia of Genes and Genomes) pathway enrichment analysis (Figure 4C,D). The analysis revealed that, compared to tramadol, *O*-desmethyltramadol treatment in MDA-MB-231 cells significantly altered the expression of genes enriched in several key signaling pathways, including the TNF (tumor necrosis factor) signaling pathway, Th1 (T helper cell type 1) and Th2 (T helper cell type 2) cell differentiation, Th17 (T helper 17) cell differentiation, Notch signaling pathway, IL-17 (interleukin-17) signaling pathway, and ECM (extracellular matrix)-receptor interaction. These pathways are known to play crucial roles in immune regulation, inflammation, and cellular communication, indicating that *O*-desmethyltramadol may modulate breast cancer cell survival and death by influencing immune responses, inflammatory processes, and interactions within the tumor microenvironment (Figure 4C). The enrichment of immune-related pathways suggests a potential involvement of *O*-desmethyltramadol in altering the inflammatory landscape of MDA-MB-231 cells, which are known to be triple-negative and highly aggressive. In contrast, compared to tramadol, *O*-desmethyltramadol treatment in MCF-7 cells revealed a distinct set of differentially expressed genes enriched in pathways related to metabolic regulation, gene transcription, and cell signaling. These pathways included vitamin B6 metabolism, transcriptional mis-regulation in cancer, pyruvate metabolism, protein digestion and absorption, the mTOR (mammalian target of rapamycin) signaling pathway, the MAPK (mitogen-activated protein kinase) signaling pathway, and ECM-receptor interaction (Figure 4D). The involvement of metabolic and transcriptional regulation pathways suggests that, compared to tramadol, *O*-desmethyltramadol may affect MCF-7 cells through mechanisms related to cellular metabolism, growth signaling, and extracellular matrix remodeling. The enrichment of the mTOR and MAPK pathways, which are critical regulators of cell proliferation and survival, implies that, compared to tramadol, *O*-desmethyltramadol may influence MCF-7 cell viability through metabolic and growth-related processes rather than immune and inflammatory responses, as observed in MDA-MB-231 cells. These findings highlight the distinct molecular responses of different breast cancer subtypes to *O*-desmethyltramadol in comparison to tramadol, suggesting that the effects of these drugs on cancer cell survival and death are highly context-dependent and may vary based on the specific signaling networks active in each cell type.

Table 1 showed the differentially expressed genes in MDA-MB-231 and MCF-7 cells following treatment with tramadol or *O*-desmethyltramadol compared to the control group. In MDA-MB-231 cells, the shared gene expression changes between tramadol and *O*-desmethyltramadol include *pellino E3 ubiquitin protein ligase family member 2* (*PELI2*), *TNF superfamily member 15* (*TNFSF15*), *ENSG00000227706*, and *ENSG00000272405*. In MCF-7 cells, the shared differentially expressed genes are *sprouty RTK signaling antagonist 4* (*SPRY4*), *H19 imprinted maternally expressed transcript* (*H19*), *keratin 6A* (*KRT6A*), and *interleukin 1 beta* (*IL1B*).

**Figure 5:**
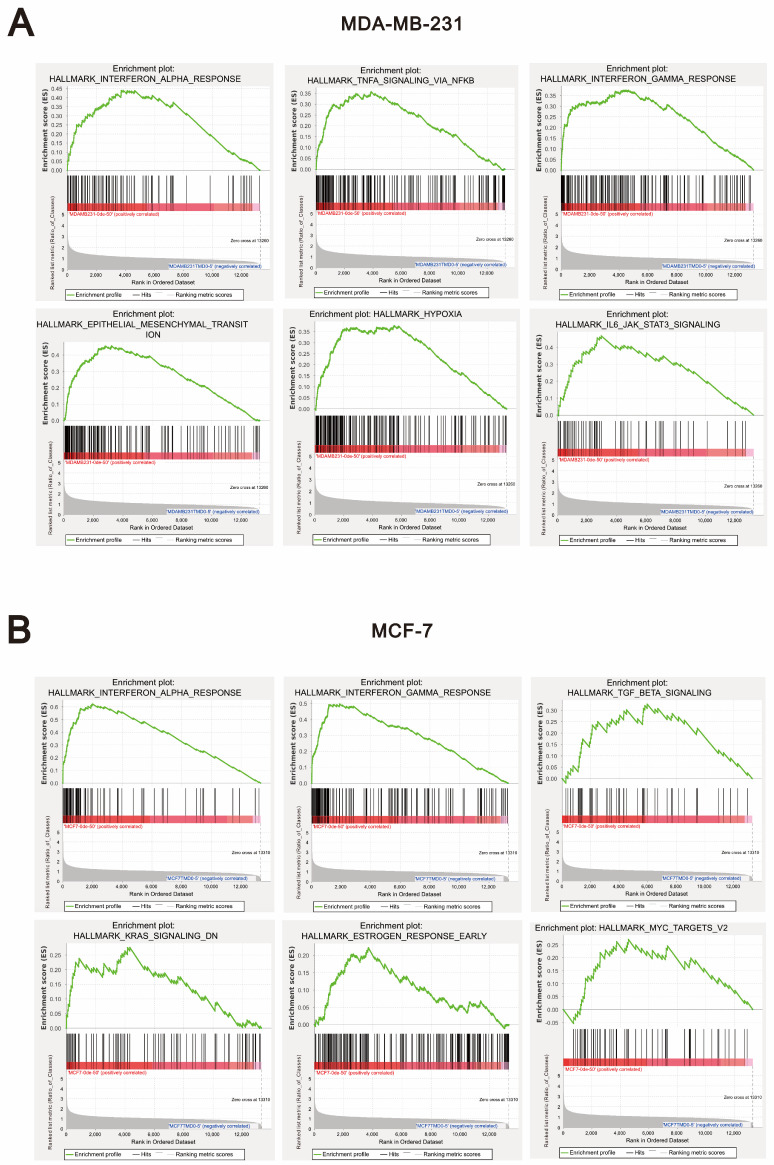
GSEA pathway enrichment analysis in breast cancer cells following *O*-desmethyltramadol treatment compared to tramadol. (**A**) Gene expression changes in MDA-MB-231 cells following treatment with 0.05 mg/mL *O*-desmethyltramadol compared to 0.5 mg/mL tramadol are associated with pathways such as the TNF signaling pathway, Th1 and Th2 cell differentiation, Th17 cell differentiation, Notch signaling pathway, IL-17 signaling pathway, and ECM-receptor interaction. (**B**) Gene expression changes in MCF-7 cells following treatment with 0.05 mg/mL *O*-desmethyltramadol compared to 0.5 mg/mL tramadol are associated with pathways such as TGF-beta signaling, KRAS-signaling-DN, early estrogen response, and MYC targets.

Next, we performed Gene Set Enrichment Analysis (GSEA) to comprehensively compare the biological responses induced by *O*-desmethyltramadol and tramadol in breast cancer cells. This analysis allowed us to identify specific gene sets and pathways that were differentially regulated by *O*-desmethyltramadol and tramadol, providing further insight into their distinct mechanisms of action. The results of the GSEA analysis revealed that, in both MDA-MB-231 and MCF-7 cells, *O*-desmethyltramadol, compared to tramadol, was significantly enriched in the interferon-alpha response and interferon-gamma response pathways (Figure 5A,B). These findings suggest that *O*-desmethyltramadol may have a stronger impact on immune-related signaling compared to tramadol. However, there were notable differences between the two breast cancer cell lines. In MDA-MB-231 cells, *O*-desmethyltramadol exhibited significant enrichment in pathways associated with TNF-alpha signaling via NF-κB (nuclear factor kappa B), epithelial-mesenchymal transition (EMT), hypoxia, and IL-6 (interleukin 6)/JAK (Janus kinases)/STAT3 (signal transducer and activator of transcription 3) signaling when compared to tramadol (Figure 5A). These pathways are known to play key roles in inflammation and the tumor microenvironment, indicating that *O*-desmethyltramadol may influence these aggressive triple-negative breast cancer cells through pro-inflammatory and hypoxia-related mechanisms. Conversely, in MCF-7 cells, *O*-desmethyltramadol was significantly enriched in pathways related to TGF-beta (transforming growth factor-beta) signaling, KRAS (Kirsten rat sarcoma viral oncogene homolog) signaling downregulation (KRAS-signaling-DN), early estrogen response, and MYC targets when compared to tramadol (Figure 5B). These pathways are closely associated with hormone receptor signaling, oncogenic transcriptional regulation, and cellular proliferation, suggesting that *O*-desmethyltramadol may exert its effects in hormone receptor-positive breast cancer cells by modulating growth factor signaling and oncogene regulation. These findings highlight the differential impact of *O*-desmethyltramadol on distinct breast cancer subtypes, suggesting that its effects are highly context-dependent and influenced by the molecular characteristics of each cell type.

### 2.4. The Differential Effects of O-Desmethyltramadol and Tramadol in MDA-MB-231 and MCF-7 Cells: Identifying Cell Type-Specific Responses

**Figure 6:**
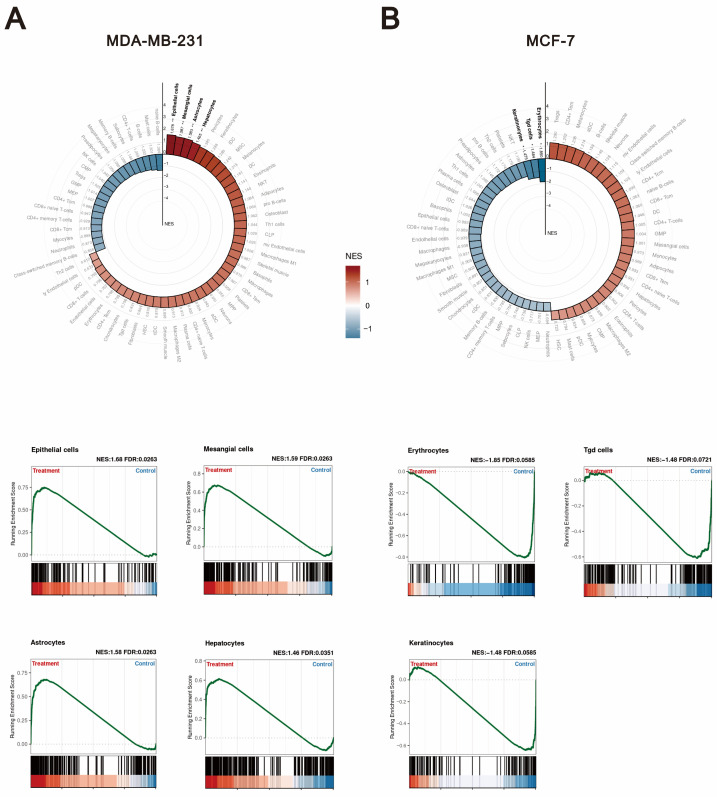
Cell type-specific responses to *O*-desmethyltramadol versus tramadol in breast cancer cells. (**A**) Cell type-specific responses in MDA-MB-231 cells following treatment with 0.05 mg/mL *O*-desmethyltramadol compared to 0.5 mg/mL tramadol are linked to epithelial cells, mesangial cells, astrocytes, and hepatocytes. (**B**) Cell type-specific responses in MCF-7 cells following treatment with 0.05 mg/mL *O*-desmethyltramadol compared to 0.5 mg/mL tramadol are associated with erythrocytes, Tgd cells, and keratinocytes.

Based on the above findings, we next sought to investigate which specific cell types are associated with the differential effects of *O*-desmethyltramadol compared to tramadol in MDA-MB-231 and MCF-7 cells. The purpose of this analysis was to determine whether *O*-desmethyltramadol influences the tumor microenvironment differently from tramadol by modulating interactions with distinct cell types. We utilized two advanced bioinformatics approaches: xCell signatures and GSEA. xCell signatures perform cell type enrichment analysis based on gene expression profiles, enabling the deconvolution of complex tissue samples into their constituent cell types [18]. This provides a detailed map of the cellular landscape. By estimating the relative abundance of various cell types using predefined gene signatures, xCell signatures offer insights into the immune and stromal components within tissues. These insights help us better understand how *O*-desmethyltramadol may influence cancer progression, immune modulation, and intercellular communication across different breast cancer subtypes. In the analysis of MDA-MB-231 cells, the differential effects of *O*-desmethyltramadol compared to tramadol were found to be statistically significant in several cell types, including epithelial cells, mesangial cells, astrocytes, and hepatocytes (Figure 6A). This suggests that *O*-desmethyltramadol may influence these specific cell types within the tumor microenvironment, potentially affecting processes such as inflammation, tissue remodeling, and cellular communication. On the other hand, in MCF-7 cells, the analysis revealed that erythrocytes, Tgd cells, and keratinocytes showed significant changes in response to *O*-desmethyltramadol treatment compared to tramadol (Figure 6B). This indicated that the effects of *O*-desmethyltramadol on MCF-7 cells may be associated with alterations in hematopoietic and skin-related cell populations, which could impact cell proliferation, differentiation, and immune responses. These findings highlight the complex and cell type-specific nature of the responses to *O*-desmethyltramadol, suggesting that its mechanisms of action differ not only between breast cancer subtypes but also in terms of the broader cellular context within the tumor microenvironment.

### 2.5. Shared Differentially Expressed Genes Regulated by O-Desmethyltramadol Compared to Tramadol in Breast Cancer Cells

**Figure 7:**
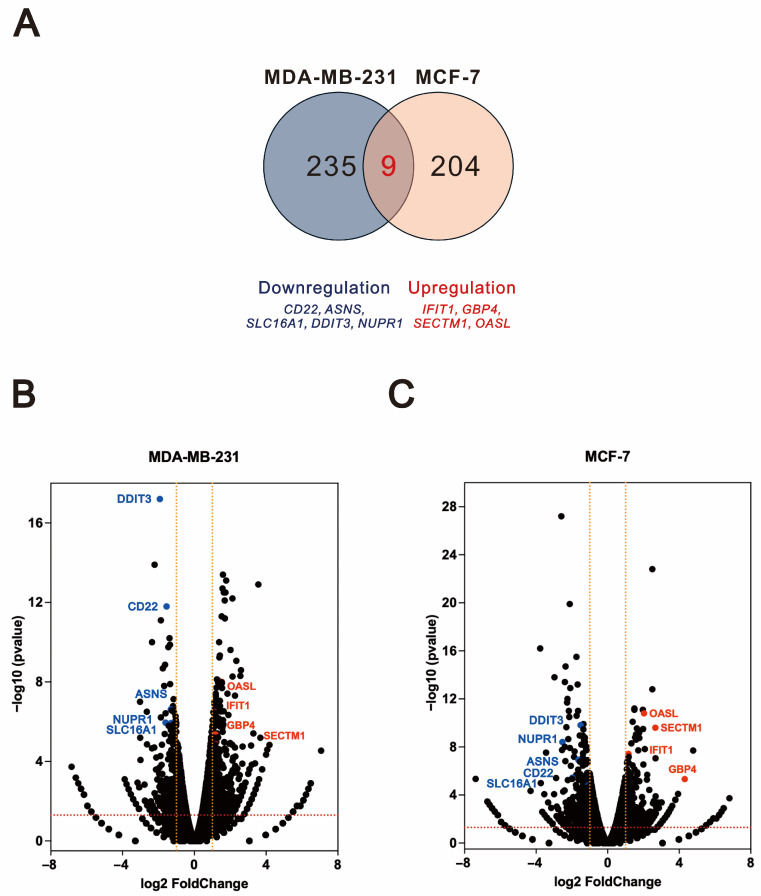
Impact of *O*-desmethyltramadol compared to tramadol on shared differentially expressed genes in breast cancer cells. (**A**) After treatment with tramadol and *O*-desmethyltramadol, compared to tramadol, nine shared differentially expressed genes were identified between MDA-MB-231 and MCF-7 cells. (**B**,**C**) The distribution of these 9 differentially expressed genes in the volcano plots of MDA-MB-231 and MCF-7 cells.

As shown in Figure 7A, the Venn diagram illustrated that nine genes were differentially expressed in both MDA-MB-231 and MCF-7 cells following treatment, while 235 and 204 genes were uniquely differentially expressed in MDA-MB-231 and MCF-7 cells, respectively. Our analysis revealed that, compared to tramadol, *O*-desmethyltramadol consistently regulated the expression of nine genes in both MCF-7 and MDA-MB-231 cells. Specifically, four genes—*interferon induced protein with tetratricopeptide repeats 1* (*IFIT1*), *guanylate binding protein 4 (GBP4), secreted and transmembrane protein 1* (*SECTM1*), and *2′-5′-oligoadenylate synthetase like* (*OASL*)—were upregulated, while five genes—*cluster of differentiation-22* (*CD22*), *asparagine synthetase* (*ASNS*), *solute carrier family 16 member 1* (*SLC16A1*), *DDIT3*, and *nuclear protein 1* (*NUPR1*)—were downregulated in MDA-MB-231 (Figure 7B) and MCF-7 (Figure 7C) cells. Notably, *DDIT3*, a key regulator of ER stress and cellular apoptosis, was induced by both *O*-desmethyltramadol and tramadol in MCF-7 and MDA-MB-231 cells; however, its induction was less pronounced with *O*-desmethyltramadol, suggesting a potential difference in the regulation of stress-related pathways in breast cancer cells.

## 3. Discussion

Our study provides novel insights into the anti-cancer effects of tramadol and its primary metabolite, *O*-desmethyltramadol, on breast cancer cells. We demonstrated that *O*-desmethyltramadol exhibited a significantly stronger cytotoxic effect than tramadol in both MDA-MB-231 and MCF-7 cells. Furthermore, the addition of the μ-opioid receptor antagonist alvimopan did not reverse the cytotoxic effects of either tramadol or *O*-desmethyltramadol, supporting the hypothesis that their anti-cancer effects are mediated through pathways other than opioid receptor activation. We also observed that *O*-desmethyltramadol induced ER stress like tramadol in breast cancer cells. Notably, the extent of ER stress induction varied between different breast cancer cell lines. MCF-7 cells exhibited a stronger response to ER stress than MDA-MB-231 cells, which may contribute to their increased sensitivity to tramadol-induced cytotoxicity. This finding aligns with previous studies, indicating that ER stress plays a crucial role in modulating cancer cell death and survival.

Our RNA-seq analysis further revealed that *O*-desmethyltramadol exerts distinct effects on different breast cancer subtypes by modulating key signaling pathways. In MDA-MB-231 cells, *O*-desmethyltramadol influenced immune-related and inflammatory pathways, including TNF signaling, Th1 and Th2 differentiation, and IL-17 signaling. These findings suggest that *O*-desmethyltramadol may exert its effects by altering the tumor microenvironment and immune response in aggressive breast cancer subtypes. In contrast, in MCF-7 cells, *O*-desmethyltramadol primarily affected metabolic and transcriptional regulatory pathways, such as mTOR signaling and MAPK signaling, which are essential for cell proliferation and survival. These differences indicate that *O*-desmethyltramadol may target distinct molecular mechanisms depending on the breast cancer subtype. The tumor microenvironment plays a critical role in tumor initiation and progression. Notably, it varies significantly across breast cancer subtypes due to distinct quantitative and qualitative differences in immune cell populations [19]. These differences contribute to the intricate and sometimes contradictory roles of immune components, which can either suppress tumor growth or facilitate its progression [20]. Immune-promoting factors are associated with a favorable prognosis and include the presence of functional CD4+ (cluster of differentiation 4) and CD8+ (cluster of differentiation 8) T cells, B cells, and plasma cells within tertiary lymphoid structures, as well as natural killer cells, dendritic cells, and FOLR2+ (folate receptor beta) macrophages, which exhibit high expression of MHC-II (major histocompatibility complex class II) [21,22,23]. Conversely, immune-suppressive factors correlate with a poorer prognosis and include regulatory T cells, neutrophils, and TREM2+ (triggering receptor expressed on myeloid cells 2) macrophages, which are characterized by reduced MHC-II expression [24,25]. In addition to immune cells, non-immune components of the tumor microenvironment, such as cancer-associated fibroblasts and adipocytes, can influence tumor immunity by secreting various growth factors and cytokines [26]. The balance between these immune cell populations determines the immunogenicity of the tumor, and ultimately influences disease outcomes. TNBC has traditionally been regarded as the most immunogenic breast cancer subtype [27]. However, emerging evidence suggests that HER2 (human epidermal growth factor receptor 2)-amplified and high-grade hormone receptor+ luminal B tumors also exhibit a relatively high abundance of tumor-infiltrating lymphocytes, which display elevated immune checkpoint expression compared to luminal A tumors—often characterized as an immune desert [28]. Tumor-associated macrophages, both immunosuppressive and immune-stimulating, are present across all four breast cancer subtypes, though they are less frequent in luminal A tumors [29].

Notably, our findings indicate that the genes upregulated by *O*-desmethyltramadol in both breast cancer cell lines—*IFIT1*, *GBP4*, *SECTM1*, and *OASL*—are regulated by interferon. IFIT1 has been associated with increased cellular sensitivity to radiation, which may contribute to improved local regional control in early-stage, node-negative breast cancer patients. This effect is likely due to its ability to inhibit translation, thereby limiting cellular replication and growth [30]. Similarly, GBP4, an interferon- and cytokine-induced gene, has been recognized for its functional relevance in various human cancers. Emerging evidence suggests that GBP4 serves as a robust pan-cancer biomarker for evaluating tumor immunological characteristics and predicting therapeutic responses [31]. Additionally, SECTM1 expression has been found to be elevated in tumors from patients who exhibit strong immunotherapeutic responses across multiple cancer types. SECTM1 is upregulated by IFN-γ (interferon-gamma)/STAT1 (signal transducer and activator of transcription 1) signaling in immuno-hot tumors, further highlighting its role in tumor-immune interactions [32]. Conversely, in both breast cancer cell lines, *O*-desmethyltramadol consistently downregulated the expression of *SLC16A1*, *CD22*, *NUPR1*, *ASNS*, and *DDIT3*. Among them, *SLC16A1* plays a crucial role in lactate transport, which is essential for tumor metabolism. Studies have shown that inhibiting or knocking down *SLC16A1* in mixed cancer cell-fibroblast xenografts in mice can delay tumor growth [33]. *CD22* was one of the most differentially expressed genes in lymph node metastases of patients with metastatic breast cancer compared to primary breast tumors. Notably, *CD22* mRNA levels were higher in lymph node metastases than in primary tumors. Moreover, its expression in primary breast tumors was significantly associated with recurrence-free survival, with a stronger correlation observed in lymph node-positive patients than in lymph node-negative patients [34]. Similarly, *NUPR1* has been found to be highly expressed in breast cancer cells. Its depletion induces premature senescence in vitro and suppresses tumor growth in vivo, highlighting its potential role in cancer progression and therapeutic targeting [35]. *ASNS* has been implicated in breast cancer tumorigenesis, as its reduced expression inhibits proliferation and induces cell cycle arrest. High levels of *ASNS* are associated with poor prognosis in breast cancer [36,37]. Finally, the expression pattern of *DDIT3* in breast cancer correlates with clinical parameters, increasing with advanced stages and suggesting its involvement in tumor progression. High *DDIT3* expression has been linked to greater invasiveness and metastatic potential, both of which are associated with poorer prognosis [38,39]. These results highlight the potential of *O*-desmethyltramadol in influencing breast cancer biology, warranting further investigation into its therapeutic implications.

The concentrations of tramadol and *O*-desmethyltramadol used in this study were chosen based on both reported therapeutic plasma levels [40] and the need to elicit measurable biological responses in a simplified cell culture system. This practice is based on the rationale that in vitro systems generally need higher extracellular drug levels to induce measurable cellular responses, including cytotoxicity, compared to the concentrations associated with adverse effects in vivo [41]. To this end, we employed a concentration range (12.5–100 μg/mL) to observe dose-dependent responses, calculate IC50 values, and compare the relative cytotoxicity of tramadol and *O*-desmethyltramadol. Future studies could explore alternative delivery strategies, such as nanoparticle-mediated drug delivery or intratumoral injection, to locally achieve therapeutically relevant concentrations of *O*-desmethyltramadol within the tumor microenvironment.

The clinical use of tramadol in cancer patients for pain management raises important considerations regarding the systemic accumulation of its active metabolite, *O*-desmethyltramadol. Due to interindividual differences in CYP2D6 enzyme activity, which is responsible for the metabolic conversion of tramadol to *O*-desmethyltramadol, some patients may experience higher systemic exposure to *O*-desmethyltramadol. This could be particularly relevant in long-term use or in individuals classified as ultra-rapid metabolizers. Given our findings that *O*-desmethyltramadol exhibits more potent cytotoxic effects on breast cancer cells than tramadol, it is plausible that sustained or elevated levels of this metabolite could influence tumor behavior or interfere with cancer progression. While the in vitro concentrations used in this study exceed typical plasma levels observed in patients, our data suggests that even modest increases in *O*-desmethyltramadol concentrations might have biological relevance in tumor microenvironments. These findings underscore the need for further pharmacokinetic and pharmacodynamic studies to evaluate the potential impact of *O*-desmethyltramadol accumulation in oncology settings, particularly in patients undergoing prolonged tramadol therapy.

## 4. Materials and Methods

### 4.1. Cell Culture and Reagents

MDA-MB-231 (HTB-26™) cells were purchased from American Type Culture Collection (Manassas, VA, USA). MCF-7 (BCRC-60436) cells were purchased from the Bioresource Collection and Research Center (Hsinchu, Taiwan). MDA-MB-231 cells were cultured in Dulbecco’s modified Eagle’s medium (DMEM) supplemented with 10% FBS and 1% penicillin–streptomycin. MCF-7 cells were cultured in minimum essential medium (MEM) supplemented with 2 mM L-glutamine and Earle’s balanced salts, which contained 1.5 g/L sodium bicarbonate, 0.1 mM nonessential amino acids, 1.0 mM sodium pyruvate, 10% fetal bovine serum (FBS), and 1% penicillin–streptomycin (Thermo Fisher Scientific, Waltham, MA, USA). Tramadol was obtained from Sigma Aldrich (Sigma Aldrich; St. Louis, MO, USA). *O*-desmethyltramadol was obtained from LGC Standards (GmbH, Wesel, Germany).

### 4.2. Cell Viability and Cytotoxicity Analysis

Cells (5 × 10^3^ per well) were seeded into 96-well plates and incubated overnight at 37 °C in a 5% CO_2_ atmosphere. The next day, the cells were treated with varying concentrations of tramadol or *O*-desmethyltramadol for 48 h. Cell viability was assessed using the CellTiter-Glo^®^ Assay (Promega, Madison, WI, USA), and luminescence signals were measured with a Varioskan LUX plate reader (Thermo Fisher Scientific, Vantaa, Finland).

### 4.3. Western Blot

After treatment with *O*-desmethyltramadol, the cells were lysed with radioimmunoprecipitation assay (RIPA) buffer to extract proteins. The protein concentration was measured with a DC protein assay, and 30 µg of protein was loaded onto an SDS-PAGE gel. Following electrophoresis, the proteins were transferred onto PVDF membranes, which were then blocked with 5% nonfat milk in TBST for 1 h. The membranes were incubated with primary antibodies overnight at 4 °C with gentle shaking, followed by incubation with secondary antibodies for 1 h at room temperature. Primary antibodies against β-actin were sourced from Santa Cruz Biotechnology (Santa Cruz, CA, USA), while those against p-eIF2α, eIF2α, ATF4, and CHOP were obtained from Cell Signaling Technology (Danvers, MA, USA).

### 4.4. RNA Isolation, Quantitative Polymerase Chain Reaction (qPCR), and RNA-seq

For RNA isolation, cells were washed twice with PBS and lysed with TRI reagent (Sigma Aldrich) according to the manufacturer’s protocol, and then, 1 μg of total RNA was reverse transcribed using MMLV reverse transcriptase (Epicenter Biotechnologies, Madison, WI, USA) at 42 °C for 60 min. The PCR primers used were as follows: *ATF4*-F, 5′-TTCTCCAGCGACAAGGCTAAGG-3′ and *ATF4*-R, 5′-CTCCAACATCCAATCTGTCCCG-3′; *CHAC-1*-F, 5′-CCTGAAGTACCTGAATGTGCGAGA-3′ and *CHAC-1*-R, 5′-GCAGCAAGTATTCAAGGTTGTGGC-3′; *DDIT3*-F, 5′-GGAAACAGAGTGGTCATTCCC-3′ and *DDIT3*-R, 5′-CTGCTTGAGCCGTTCATTCTC-3′; *ACTB*-F, 5′-ACAGGAAGTCCCTTGCCATC-3′ and *ACTB*-R, 5′-CAGTGTACAGGTAAGCCCTGG-3′.

For RNA-seq, 1 μg of total RNA was used to prepare the library. Poly(A) mRNA was isolated using Oligo(dT) beads, followed by fragmentation with divalent cations at high temperature. Random primers were used for priming, and first- and second-strand cDNA synthesis was performed. The purified double-stranded cDNA underwent end repair and dA-tailing in a single reaction, followed by adaptor ligation using T-A ligation. Adaptor-ligated DNA was size-selected with DNA Clean Beads. Each sample was amplified using PCR with P5 and P7 primers, and the PCR products were validated. Libraries with unique indices were multiplexed and sequenced on an Illumina HiSeq, NovaSeq, or MiSeq 2000 system in a 2 × 150 paired-end configuration, following the manufacturer’s protocol.

### 4.5. Differential Expression Analysis and Enrichment Analysis

Transcript sequences in FASTA format were first generated from the reference GFF annotation file and properly indexed. Using this reference, gene and isoform expression levels were quantified from the paired-end clean reads with HTSeq (v0.6.1). Differential expression analysis was performed using the DESeq2 Bioconductor package (v1.6.3), which incorporates data-driven prior distributions for the estimation of dispersion and log2 fold changes. Genes with an adjusted *p*-value (Padj) ≤ 0.05 were considered significantly differentially expressed. To further validate differential expression, we also utilized EdgeR (v3.4.6), applying a threshold of log2 fold change > 1 (i.e., fold change > 2) and false discovery rate (FDR, Q-value or Padj) < 0.05 to identify significant genes.

Functional enrichment analysis of differentially expressed genes was conducted using the Kyoto Encyclopedia of Genes and Genomes (KEGG) database (accessed on 8 January 2025). In-house scripts were employed to map these genes to KEGG pathways. Enrichment was quantified using three parameters: RichFactor, Q-value, and the number of genes mapped to each pathway. RichFactor represents the ratio of differentially expressed genes to the total number of genes in each pathway—a higher RichFactor indicates greater enrichment. Q-value, the *p*-value adjusted for multiple hypothesis testing, ranges from 0 to 1, with values closer to 0 denoting more significant enrichment. For visualization, the top 30 enriched pathways (ranked by Q-value) were selected; if fewer than 30 pathways met the criteria, all were displayed. Gene Set Enrichment Analysis (GSEA) was performed to explore potential molecular mechanisms through which *O*-desmethyltramadol, relative to tramadol, may regulate tumor progression. The “h.all.v2024.1.Hs.symbols.gmt” hallmark gene set was used to identify significantly enriched pathways between the two groups. For xCell signature analysis, three cell type-specific gene sets described by Aran et al. were integrated and analyzed using the GSEA function within the clusterProfiler package (v4.10.0) [42,43]. The enrichment results for each cell type were visualized individually as radial plots generated with ggplot2 (v3.4.0), providing insight into cellular composition and immune landscape differences between treatment groups.

### 4.6. GSH/GSSG Measurement

To determine the GSH/GSSG ratio, the GSH/GSSG-Glo Assay (Promega) was conducted according to the manufacturer’s instructions. Briefly, after the specified treatment period, cells in a 96-well plate were rinsed with PBS and treated with 50 μL of either total GSH lysis reagent (for total GSH quantification) or oxidized GSH lysis reagent (for GSSG measurement). Next, 50 μL of Luciferin Generation Reagent was added to each well and incubated for 30 min. Subsequently, 100 μL of Luciferin Detection Reagent was applied, followed by an additional 15-min incubation before luminescence was recorded. The GSH/GSSG ratio was calculated from the luminescence values, expressed in relative light units (RLU), using the formula: (total GSH RLU − GSSG RLU)/(GSSG RLU/2).

### 4.7. ROS Measurement

Intracellular ROS levels were assessed using DCFH-DA staining. After treatment with tramadol or *O*-desmethyltramadol, cells were washed twice with PBS, and then incubated with 10 μM DCFH-DA at 37 °C for 30 min in the dark. Subsequently, the cells were washed with PBS and analyzed using a FACSCalibur flow cytometer in conjunction with Cell Quest Pro software (v5.1).

### 4.8. Statistics

Bar graphs include individual data points to represent biological replicate numbers. The figure legends for the line graphs specify the number of biological replicates. The data are expressed as the mean ± standard error of the mean (SEM). Statistical significance was determined using GraphPad software with one-way ANOVA and Tukey’s multiple comparisons, two-way ANOVA with Dunnett’s multiple comparisons, or a two-tailed Student’s *t* test, as appropriate. The error bars on the graphs represent the SEM, and the significance between samples is denoted as * *p* < 0.05, ** *p* < 0.01, *** *p* < 0.001, and **** *p* < 0.0001.

## 5. Conclusions

Our study provides compelling evidence that *O*-desmethyltramadol exhibits superior anti-cancer effects compared to tramadol through distinct, non-opioid receptor-mediated mechanisms. Our RNA-seq analysis further revealed that *O*-desmethyltramadol exerts distinct effects on different breast cancer subtypes by modulating key signaling pathways. These findings pave the way for future research to explore the therapeutic potential of *O*-desmethyltramadol as an adjunct in breast cancer treatment. Given the potent in vitro anti-cancer effects of *O*-desmethyltramadol, further in vivo studies are warranted to assess its therapeutic potential and safety profile within the tumor microenvironment. Additionally, these findings raise important considerations regarding the long-term use of tramadol in cancer patients, as sustained exposure to its active metabolite may inadvertently influence tumor progression.

## Figures and Tables

**Table 1:** The differential and shared differentially expressed genes between tramadol and *O*-desmethyltramadol in breast cancer cells.

MDA-MB-231. control vs. tramadol differentially expressed genes	*SPX*, *CLCA3P*, *ENSG00000283265*, *ENSG00000272468*, *PELI2*, *HEY1*, *ENSG00000232053*, *OLAH*, *SLC16A12*, *PSMD10P2*, *LINC00632*, *LINC02392*, *CHRM3-AS2*, *PLA2G4C*, *PINLYP*, *ENSG00000253227*, *NGF*, *ATP6V0D2*, *MMP3*, *NMRAL2P*, *SLC16A1*, *TNFSF15*, *LNCOG,SUN3*, *NCF2*, *XIRP2*, *NUPR1*, *ENSG00000236453*, *DDIT3*, *LAMA1*, *MAL2*, *BCAN*, *LINC01293*, *ENSG00000237596*, *LURAP1L-AS1*, *LINC03002*, *FLI1,MXD1*, *LINC01291*, *GAPLINC*, *TM4SF19*, *MMP1*, *ENSG00000286975*, *PTPRR*, *DCLK1*, *B3GALT5*, *TM4SF19-AS1*, *DLGAP1-AS2*, *AK5*, *ENSG00000285108*, *TOX*, *PTPN22*, *CREBRF*, *RCAN1*, *IL24*, *HDAC9*, *RASGRP3*, *ENSG00000266401*, *ENSG00000227706*, *TMEM236*, *MPP4*, *DNAJB9*, *CXCL2*, *LAMP3*, *TRIB3*, *ENSG00000272405*, *VNN1*, *RGMB-AS1*, *PARM1*, *SEC24A*, *DOCK9*, *ENSG00000260604*, *IGFN1*, *SP140*, *PPARGC1A*, *HERPUD1*, *LRATD2*, *PPP1R15A*, *HKDC1*, *FGF1*, *EVI2A*, *SEC24D*, *GFPT1*, *ENSG00000287023*, *SESN2*, *BCAN-AS1*, *COBLL1*, *OSBP2*, *ZNF114*, *GEM*, *AADACP1*, *AGRN*, *FAM20C*, *COL7A1*, *LAMA5*, *ADAMTS15*, *PRKAR1B*, *SNRPA*, *MARCKSL1*, *C1R*, *BCAM*, *UCP2*, *FCGBP*, *SREBF1*, *SCNN1A*, *ID1*, *SIGIRR*, *IGFBP6*, *VWA1*, *SHROOM1*, *ABCA1*, *SAA1*, *MXD3*, *PDE7B*, *CEMIP*, *BCL3*, *U2AF1*, *TNFSF10*, *TYMSOS*, *PFN1P1*, *CEBPD*, *SAA2*, *ENSG00000281181*, *AQP3*, *LMNTD2-AS1*, *TFAP2A-AS1*, *SOCS1*, *ST7-AS1*, *C1QL1*, *ALG1L5P*, *ZFP91-CNTF*
MDA-MB-231 control vs. *O*-desmethyltramadol differentially expressed genes	*IFITM10*, *HSPA6*, *ENSG00000272825*, *F12*, *DHRS2*, *PELI2*, *ANGPT1*, *HSD11B1*, *DPP4*, *ITGB2-AS1*, *PCDH7*, *CD44-DT*, *ENSG00000227496*, *CTSW*, *SCN9A*, *TNFSF15*, *KYNU*, *SLCO4C1*, *TMEM158*, *SHOC1*, *ENSG00000272405*, *ENSG00000227706*, *SLITRK4*, *TMEM45A*
MDA-MB-231 tramadol and *O*-desmethyltramadol shared differentially expressed genes	*PELI2*, *TNFSF15*, *ENSG00000227706*, *ENSG00000272405*
MCF-7 control vs. tramadol differentially expressed genes	*PSG5*, *TSKU-AS1*, *LRRC15*, *NUPR1*, *SPRY4*, *BEST1*, *ETV4*, *GUSBP13*, *ENSG00000256433*, *DUSP6*, *SLCO4C1*, *TMEM156*, *ENSG00000289223*, *ENSG00000233052*, *ETV5*, *HRK*, *ENSG00000260604*, *PSG9*, *RAP1AP*, *CLGN*, *DIO2*, *RAB3IL1*, *NIBAN1*, *STARD4*, *SHC4*, *BEX2*, *LPIN1*, *NOVA1*, *CD22*, *CLDN1*, *GPNMB*, *RRAGD*, *RASD1*, *BCAT1*, *RUNX2*, *ACSS2*, *SCD*, *SLITRK6*, *INSIG1-DT*, *KCNMA1*, *COL5A2*, *SCNN1A*, *GPX8*, *DDIT3*, *TNIK*, *ICAM1*, *RIPOR3*, *NEU1*, *MVK*, *TGFBI*, *ULBP1*, *SLC7A11*, *FBN1*, *MAFF*, *HMGCS1*, *MSMO1*, *INSIG1*, *PFKFB2*, *NPC2*, *MMAB*, *LIF*, *SIGLEC15*, *ACSS1*, *PSAT1*, *DUSP4*, *WLS*, *BHLHE40*, *PLA2G4C*, *ERRFI1*, *LDLR*, *FADS2*, *BRI3*, *ACSL1*, *QPRT*, *CAPG*, *MUC16*, *MAFB*, *ST8SIA4*, *PCDH19*, *XAF1*, *LINC01522*, *NKAIN1*, *PLEKHN1*, *ZDHHC22*, *ARHGEF37*, *RNF32-AS1*, *AFAP1L2*, *OASL*, *CPA6*, *COL4A4*, *OAS2*, *GRHL3*, *PKP1*, *HCK*, *FGF18*, *CMPK2*, *H19*, *KRT16*, *LRP4*, *MX2*, *RSAD2*, *PCDH11X*, *GCOM1*, *MGAT4EP*, *MAB21L4*, *ZBTB47*, *A2ML1*, *GABRP*, *GNG4*, *KRT6A*, *SCNN1G*, *IL1B*
MCF-7 control vs. *O*-desmethyltramadol differentially expressed genes	*SPRY4*, *SLIT1*, *ENSG00000287315*, *ENSG00000277159*, *ENSG00000281181*, *IFIT2*, *OTULINL*, *NCAPG*, *UBE2C*, *CENPE*, *PRR11*, *DLGAP5*, *KIF15*, *HMMR*, *ATP8A2*, *SKA1*, *FAM83D*, *KIF14*, *CDKN2C*, *NEURL1B*, *APCDD1*, *CDCA3*, *IQGAP3*, *HASPIN*, *CDC25C*, *ANKRD35*, *TGM2*, *KRT13*, *H19*, *ARHGAP33*, *ANXA8*, *CCN5*, *PIF1*, *PHGDH*, *TP63*, *ALDH1L2*, *KRT6A*, *IL1B*
MCF-7 tramadol and *O*-desmethyltramadol shared differentially expressed genes	*SPRY4*, *H19*, *KRT6A*, *IL1B*

## Data Availability

The datasets generated and/or analyzed during this study are available from the corresponding author upon reasonable request. Additionally, our RNA-seq data has been deposited in the NCBI Gene Expression Omnibus under accession number GSE292968.
